# Multimodal Data Integration Enhance Longitudinal Prediction of New-Onset Systemic Arterial Hypertension Patients with Suspected Obstructive Sleep Apnea

**DOI:** 10.31083/j.rcm2507258

**Published:** 2024-07-10

**Authors:** Yi Yang, Haibing Jiang, Haitao Yang, Xiangeng Hou, Tingting Wu, Ying Pan, Xiang Xie

**Affiliations:** ^1^Xinjiang Medical University, 830011 Urumqi, Xinjiang, China; ^2^Department of Cardiology, The Fourth Affiliated Hospital of Xinjiang Medical University, 830099 Urumqi, Xinjiang, China; ^3^Department of Hypertension, The First Affiliated Hospital of Xinjiang Medical University, 830011 Urumqi, Xinjiang, China

**Keywords:** obstructive sleep apnea syndrome, major adverse cardiac and cerebrovascular events, multimodal data integration, systemic arterial hypertension, apnea-hypopnea index, triglyceride glucose index

## Abstract

**Background:**

It is crucial to accurately predict the disease progression of systemic arterial hypertension in order to determine the most effective therapeutic strategy. To achieve this, we have employed a multimodal data-integration approach to predict the longitudinal progression of new-onset systemic arterial hypertension patients with suspected obstructive sleep apnea (OSA) at the individual level.

**Methods:**

We developed and validated a predictive nomogram model that utilizes multimodal data, consisting of clinical features, laboratory tests, and sleep monitoring data. We assessed the probabilities of major adverse cardiac and cerebrovascular events (MACCEs) as scores for participants in longitudinal cohorts who have systemic arterial hypertension and suspected OSA. In this cohort study, MACCEs were considered as a composite of cardiac mortality, acute coronary syndrome and nonfatal stroke. The least absolute shrinkage and selection operator (LASSO) regression and multiple Cox regression analyses were performed to identify independent risk factors for MACCEs among these patients.

**Results:**

448 patients were randomly assigned to the training cohort while 189 were assigned to the verification cohort. Four clinical variables were enrolled in the constructed nomogram: age, diabetes mellitus, triglyceride, and apnea-hypopnea index (AHI). This model accurately predicted 2-year and 3-year MACCEs, achieving an impressive area under the receiver operating characteristic (ROC) curve of 0.885 and 0.784 in the training cohort, respectively. In the verification cohort, the performance of the nomogram model had good discriminatory power, with an area under the ROC curve of 0.847 and 0.729 for 2-year and 3-year MACCEs, respectively. The correlation between predicted and actual observed MACCEs was high, provided by a calibration plot, for training and verification cohorts.

**Conclusions:**

Our study yielded risk stratification for systemic arterial hypertension patients with suspected OSA, which can be quantified through the integration of multimodal data, thus highlighting OSA as a spectrum of disease. This prediction nomogram could be instrumental in defining the disease state and long-term clinical outcomes.

## 1. Introduction

At the point of healthcare, clinical decision support provides healthcare professionals and patients with pertinent information and well-informed recommendations [1, 2].

Detecting diseases at an early stage could greatly support precision therapy and long-term management of chronic diseases, especially for cardiovascular disease [1, 2, 3, 4, 5, 6, 7]. At present, the detection of systemic arterial hypertension allows for the implementation of preventative measures such as lifestyle adjustments and antihypertensive therapy, in order to avert the onset of cardiovascular disease [8, 9]. It must be acknowledged that systemic arterial hypertension is a multifaceted ailment with various contributing factors and diverse clinical presentations [8, 9, 10, 11, 12, 13, 14]. Variations in the quantity of known and underlying risk factors give rise to varying degrees of risk for unfavorable cardiac and cerebrovascular events after the onset of systemic arterial hypertension [11, 12, 13]. Notably, this phenotypic spectrum of systemic arterial hypertension is missed with the binary classification of blood pressure [13, 14]. Missed identification of systemic arterial hypertension patients at high risk might lead to poorer outcomes [13, 14].

Currently, obstructive sleep apnea (OSA) poses a significant threat to the health of the general population and individuals with systemic arterial hypertension in our fast-developing modern society [15]. OSA and systemic arterial hypertension share similar risk factors and pathological mechanisms, with high levels of inflammation and metabolic disturbance playing a crucial role in contributing to devastating events [16]. Given this, a binary diagnosis of systemic arterial hypertension fails to capture the complexity of the disease and does not accurately quantify its severity or the associated risk of adverse cardiac and cerebrovascular events. Epidemiological studies suggest that OSA prevalence is as high as 40% in patients with systemic arterial hypertension [15]. As a consequence, it is prudent to assess the quantitative risk of systemic arterial hypertension in individuals with OSA. Clinically, clinicians usually rely on patients’ symptoms, blood pressure readings, sleep monitoring data, and physical signs to diagnose and treat systemic arterial hypertension and sleep apnea. Previous studies have provided new insights into the integration of various clinical data types, as well as the development and validation of a clinical model for personalized prediction of systemic arterial hypertension in consecutive patients diagnosed with OSA [17]. However, little progress has been made in integrating multimodal data to enhance the longitudinal predictive capacity and accurately characterize the status of chronic disease and long-term prognostic risk of hypertensive patients with OSA. Additionally, while systemic arterial hypertension is easily detected clinically, OSA may often lead to missed or misdiagnosis, resulting in a high ratio of adverse cardiac events. Therefore, this study was conducted using multiple data sources, including sleep monitoring data, laboratory test parameters, and clinical text data, to improve the comprehensive assessment and longitudinal predictive ability of systemic arterial hypertension patients suspected OSA.

## 2. Methods

### 2.1 Study Design and Participants

Consecutive new-onset systemic arterial hypertension patients with suspected OSA were extracted from clinical data in the Xinjiang Medical University Affiliated Hospital of Traditional Chinese Medicine from January 2018 to December 2019. In our study, systemic arterial hypertension was diagnosed based on the criteria established [18]. Additionally, patients who had undergone a first diagnostic sleep via successful polysomnography were screening during their duration of hospital stay. A complete list of inclusion and exclusion criteria can be found in the **Supplementary Material**. We obtained approval from the Institutional Review Board of Xinjiang Medical University Affiliated Hospital of Traditional Chinese Medicine (No. 2022XE0103-1) for this study. As it was a retrospective study, the requirement for informed consent from patients for inclusion in the study was waived by the Institutional Review Board.

### 2.2 Sleep Monitoring Study

Each participant underwent polysomnography (Grael; Compumedics, Melbourne, VIC, Australia) to acquire the following parameters: airflow, thoraco-abdominal movements, pulse oximetry, and snore episodes. Patients who had been diagnosed with or were suspected of having OSA were identified from the sleep monitoring report. OSA was defined by the apnea-hypopnea index (AHI), which measures the number of apneas and hypopneas per hour of sleep. Hypopnea was consistently defined as either a decrease of more than 50% in breathing amplitude lasting at least 10 seconds, or a smaller decrease in amplitude lasting at least 10 seconds that was associated with either a drop in oxygen saturation of at least 3% or by arousal.

### 2.3 Common Clinical and Laboratory Assessments

On the morning following admission, peripheral venous blood with patients fasting was collected for analysis of various parameters consisting of fasting blood glucose (FBG), glycosylated hemoglobin (HbAIc), lipid levels, fibrinogen levels, D-dimer levels, liver function, kidney function, and routine blood examination parameters. Clinical text data gathered from medical records, prior medication, and self-reports was administrated to determine the baseline characteristics of the population.

### 2.4 Clinical Endpoint

Following discharge, patients were followed up either by phone or by clinical visit. The primary endpoint was the 3-year rate of major adverse cardiac and cerebrovascular events (MACCEs), defined as the composite of cardiac mortality, acute coronary syndrome, or nonfatal stroke.

### 2.5 Statistical Analysis

Statistical analysis of the study results was conducted using R software version 4.2.2 (R Foundation for Statistical Computing, Vienna, Austria) and SPSS version 23 (IBM Corp., Armonk, NY, USA). To compare the distributions of potential variables between the training and verification cohorts, variables with continuous distributions are presented as mean ± SD, otherwise, medians and interquartile ranges (IQRs) are employed. An analysis of categorical variables was conducted using a chi-square (χ^2^) test.

Multimodal data sources, containing sleep monitoring data, physiological parameters, and clinical text data were entered into a least absolute shrinkage and selection operator (LASSO) regression in the training cohort, which itself used 10-fold cross-validation to define the optimal penalization.

Potential variables were screened by LASSO regression first, followed by multivariate Cox regression analysis of the resulting significant variables. The performance of the predictive model was analyzed using previously reported methods and briefly described here [19]. To determine the model’s discriminatory ability, we calculated the Harrell’s concordance index (C-index) and the corresponding 95% CI. Concurrently, we created calibration plots to compare the predicted probabilities of MACCEs at 2 and 3 years with the observed outcomes, providing insight into the prediction model’s calibration.

## 3. Results

### 3.1 Clinical Characteristics of Patients in the Training and Verification Sets

The study retrospectively included 637 consecutive systemic arterial hypertension patients with suspected OSA. It was determined that clinical outcome events would be equally spread between a training cohort (n = 448) and a verification cohort (n = 189) by randomly assigning patients in a 7:3 ratio to the two cohorts.

Data on sleep monitoring data, laboratory test parameters, and clinical text data did not differ significantly between the training and verification cohorts (Table 1). Across both training and verification cohorts, the rate of MACCEs was similar. The average time from systemic arterial hypertension patients with suspected OSA to initial risk assessment was 35.46 months in the training cohort and 35.38 months in the verification cohort.

**Table 1.** Baseline patient characteristics and MACEs (clinical outcome) in the development and validation ${\mathrm{cohorts}}^{*}$.

Characteristic or outcome		All cohort	Development cohort	Validation cohort	*p*
		(n = 637)	(n = 448)	(n = 189)	
Male sex		460 (72.2%)	323 (72.1%)	137 (72.5%)	0.997
Age, years		54.0 [47.0; 61.0]	54.0 [47.8; 61.0]	55.0 [47.0; 62.0]	0.664
Previous CAD, n (%)		313 (49.1%)	228 (50.9%)	85 (45.0%)	0.201
Current smoking, n (%)		295 (46.3%)	207 (46.2%)	88 (46.6%)	1.000
Current alcohol drinking, n (%)		258 (40.5%)	184 (41.1%)	74 (39.2%)	0.717
Diabetes mellitus, n (%)		164 (25.7%)	107 (23.9%)	57 (30.2%)	0.120
Impaired glucose tolerance, n (%)		86 (13.5%)	65 (14.5%)	21 (11.1%)	0.308
Family history of hypertension, n (%)		243 (38.1%)	171 (38.2%)	72 (38.1%)	1.000
Non alcoholic fatty liver disease, n (%)		358 (56.2%)	263 (58.7%)	95 (50.3%)	0.061
BMI, ${\mathrm{kg/m}}^{2}$		27.9 [25.7; 31.1]	27.8 [25.8; 31.1]	28.3 [25.3; 30.9]	0.609
Abdominal girth, cm		95.0 [88.0; 103]	95.0 [87.0; 104]	95.0 [89.0; 102]	0.581
Neck circumference, cm		40.0 [38.0; 44.0]	40.0 [38.0; 44.0]	40.0 [37.0; 43.0]	0.423
Systolic blood pressure, mmHg		138 [125; 152]	139 [125; 152]	137 [128; 150]	0.891
Diastolic blood pressure, mmHg		85.0 [76.0; 94.0]	84.5 [75.0; 95.0]	85.0 [78.0; 93.0]	0.577
HbAIc		5.80 [5.49; 6.38]	5.81 [5.53; 6.34]	5.74 [5.45; 6.46]	0.449
HDL-C, mmol/L		1.23 [1.07; 1.38]	1.22 [1.06; 1.39]	1.26 [1.11; 1.37]	0.263
LDL-C, mmol/L		2.25 [1.77; 2.75]	2.29 [1.79; 2.75]	2.20 [1.74; 2.70]	0.349
Total cholesterol, mmol/L		4.27 [3.58; 4.96]	4.33 [3.61; 5.01]	4.22 [3.54; 4.87]	0.453
Fasting plasma glucose, mmol/L		5.29 [4.75; 6.22]	5.34 [4.74; 6.12]	5.18 [4.76; 6.51]	0.997
Triglyceride, mmol/L		1.65 [1.21; 2.30]	1.65 [1.23; 2.29]	1.64 [1.12; 2.36]	0.641
ApoA1, g/L		1.22 [1.11; 1.36]	1.21 [1.09; 1.36]	1.24 [1.13; 1.36]	0.197
ApoB, g/L		0.92 [0.77; 1.07]	0.92 [0.78; 1.08]	0.91 [0.75; 1.06]	0.390
Lipoprotein (a), g/L		128 [65.1; 265]	129 [65.0; 279]	128 [65.3; 243]	0.515
Creatinine, mmol/L		76.0 [65.2; 86.9]	75.5 [65.0; 87.0]	77.0 [67.0; 85.0]	0.646
Blood urea nitrogen, mmol/L		5.20 [4.40; 6.20]	5.20 [4.40; 6.20]	5.30 [4.50; 6.30]	0.328
Uric acid, µmol/L		350 (80.7)	353 (82.5)	344 (76.1)	0.173
D-Dimer, mg/L		0.50 [0.31; 0.63]	0.49 [0.31; 0.63]	0.51 [0.33; 0.62]	0.750
Fibrinogen, g/L		2.94 [2.56; 3.41]	2.94 [2.56; 3.39]	2.98 [2.58; 3.43]	0.700
Hemoglobin, g/L		150 (15.4)	150 (15.3)	150 (15.9)	0.869
White blood cells, ${\mathrm{10}}^{9}$/L		6.55 [5.62; 7.69]	6.55 [5.64; 7.65]	6.54 [5.51; 7.75]	1.000
Platelets, ${\mathrm{10}}^{9}$/L		234 [197; 272]	236 [197; 273]	229 [193; 269]	0.354
Platelet distribution width, %		12.0 [10.9; 13.4]	12.0 [10.8; 13.4]	12.1 [11.0; 13.7]	0.381
Mean platelet volume, fL		10.3 [9.69; 11.0]	10.1 [9.69; 10.9]	10.3 [9.69; 11.0]	0.204
Alanine aminotransferase, U/L		24.5 [17.9; 35.1]	24.9 [17.4; 35.2]	24.0 [18.6; 35.1]	0.803
aspartate amino transferase, U/L		21.3 [17.9; 25.9]	21.2 [18.0; 26.1]	21.4 [17.9; 25.2]	0.972
ALT/AST		1.15 [0.93; 1.41]	1.14 [0.92; 1.40]	1.16 [0.95; 1.42]	0.426
Gamma-glutamyl transferase, U/L		31.8 [21.0; 48.0]	32.0 [21.0; 48.7]	31.2 [21.7; 47.2]	0.947
ALB, g/L		41.2 [39.0; 43.4]	41.3 [38.9; 43.4]	41.0 [39.2; 43.2]	0.419
TBIL µmol/L		12.8 [10.1; 16.3]	12.8 [10.1; 16.5]	12.9 [10.2; 16.3]	0.905
Apnea hyponea index		15.2 [8.10; 27.4]	15.6 [8.50; 28.2]	15.0 [7.60; 24.9]	0.276
Average blood oxygen saturation		92.0 [90.0; 93.0]	92.0 [90.0; 93.0]	92.0 [90.0; 93.0]	0.384
Lowest blood oxygen saturation		82.0 [75.0; 85.0]	81.5 [75.0; 85.0]	82.0 [75.0; 85.0]	0.815
Lowest heart rate during sleep, beats/min		58.0 [52.0; 63.0]	57.5 [52.0; 63.0]	58.0 [52.0; 63.0]	0.671
Highest heart rate during sleep, beats/min		79.0 [73.0; 87.0]	79.0 [73.0; 88.0]	80.0 [72.0; 85.0]	0.750
Apnea maximum duration		58.0 [48.0; 68.0]	58.0 [48.0; 68.0]	58.0 [48.0; 69.0]	0.688
Mean hypopnea duration		28.0 [24.0; 33.0]	28.0 [25.0; 33.0]	29.0 [24.0; 33.0]	0.926
The longest hypopnea event duration		58.0 [46.0; 72.0]	57.0 [46.0; 70.2]	60.0 [46.0; 74.0]	0.318
MACCEs		82 (12.9%)	53 (11.8%)	29 (15.3%)	0.280
	Cardiac mortality, n (%)	11 (1.73%)	6 (1.34%)	5 (2.65%)	0.317
	Acute coronary syndrome, n (%)	62 (9.73%)	40 (8.93%)	22 (11.6%)	0.364
	Nonfatal stroke, n (%)	21 (3.30%)	14 (3.12%)	7 (3.70%)	0.896

* Values are given as n (%), mean ± SD, or median (IQR). Abbreviations: CAD, coronary artery disease; BMI, body mass index; HDL-C, high-density lipoprotein cholesterol; LDL-C, low-density lipoprotein cholesterol; MACEs, major adverse cardiac events; ApoA1, the apolipoprotein A1; ApoB, apolipoprotein B; ALT/AST, alanine aminotransferase to aspartate aminotransferase ratio; ALB, albumin; TBIL, totalbilirubin; MACCEs, major adverse cardiac and cerebrovascular events; IQR, interquartile range; HbAIc, glycosylated hemoglobin.

### 3.2 Prediction Nomogram

**Fig. 1.**
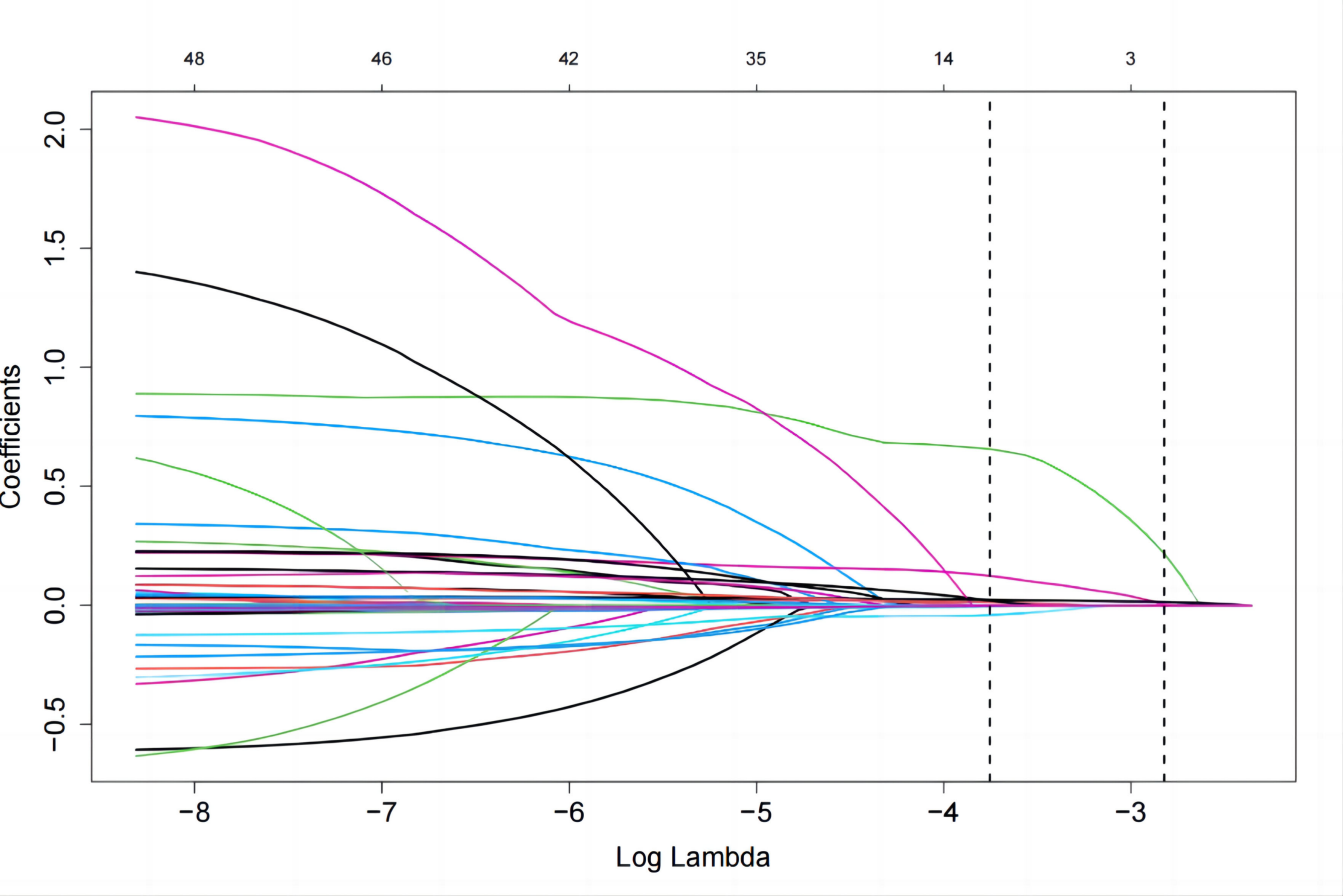
**Least absolute shrinkage and selection operator (LASSO) regression analysis for 48 variables underlying correlate with long-term major adverse cardiac and cerebrovascular events (MACCEs)**.

**Fig. 2.**
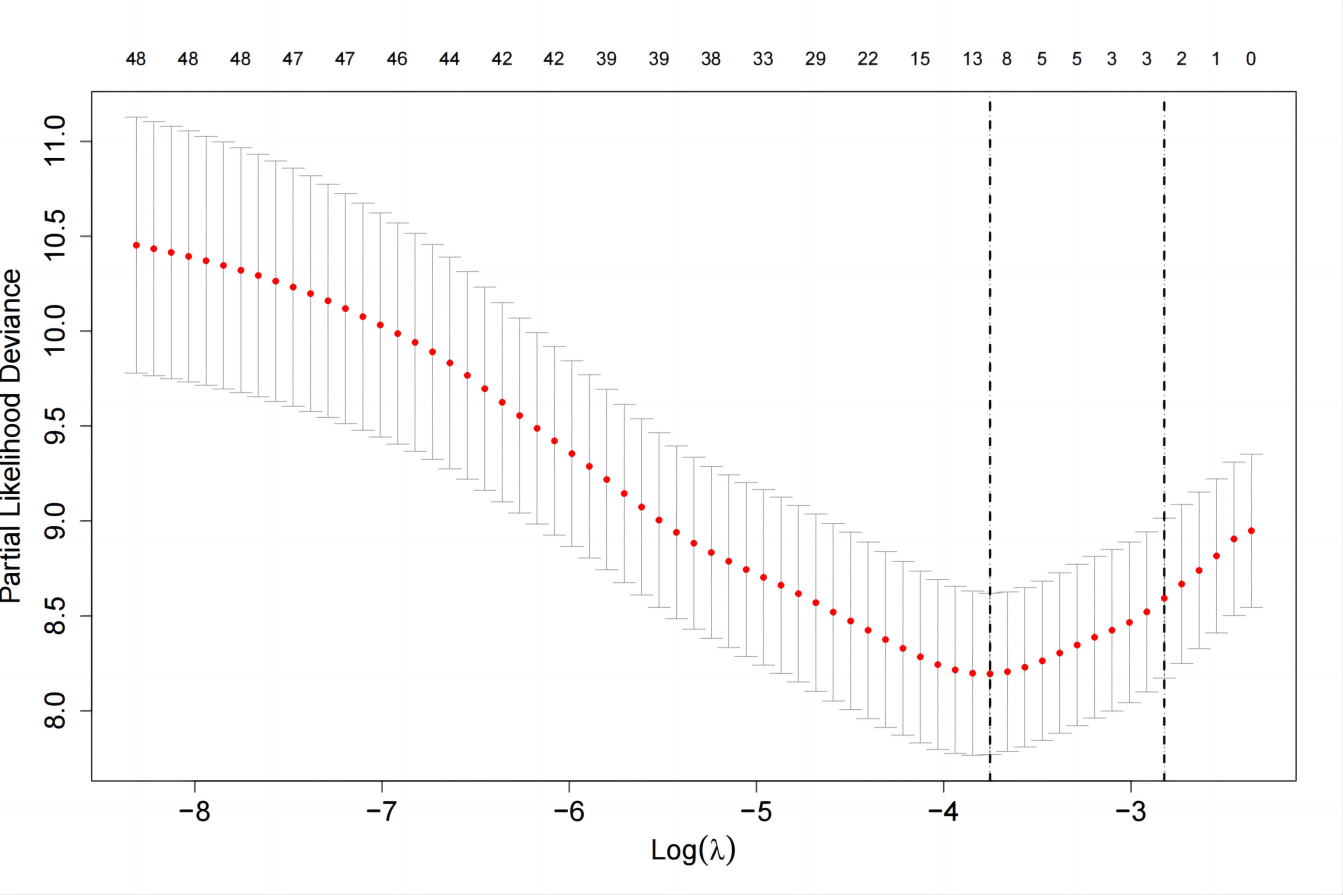
**To select the optimal tuning parameter (lambda, λ), a 10-fold cross-validation was performed**.

**Fig. 3.**
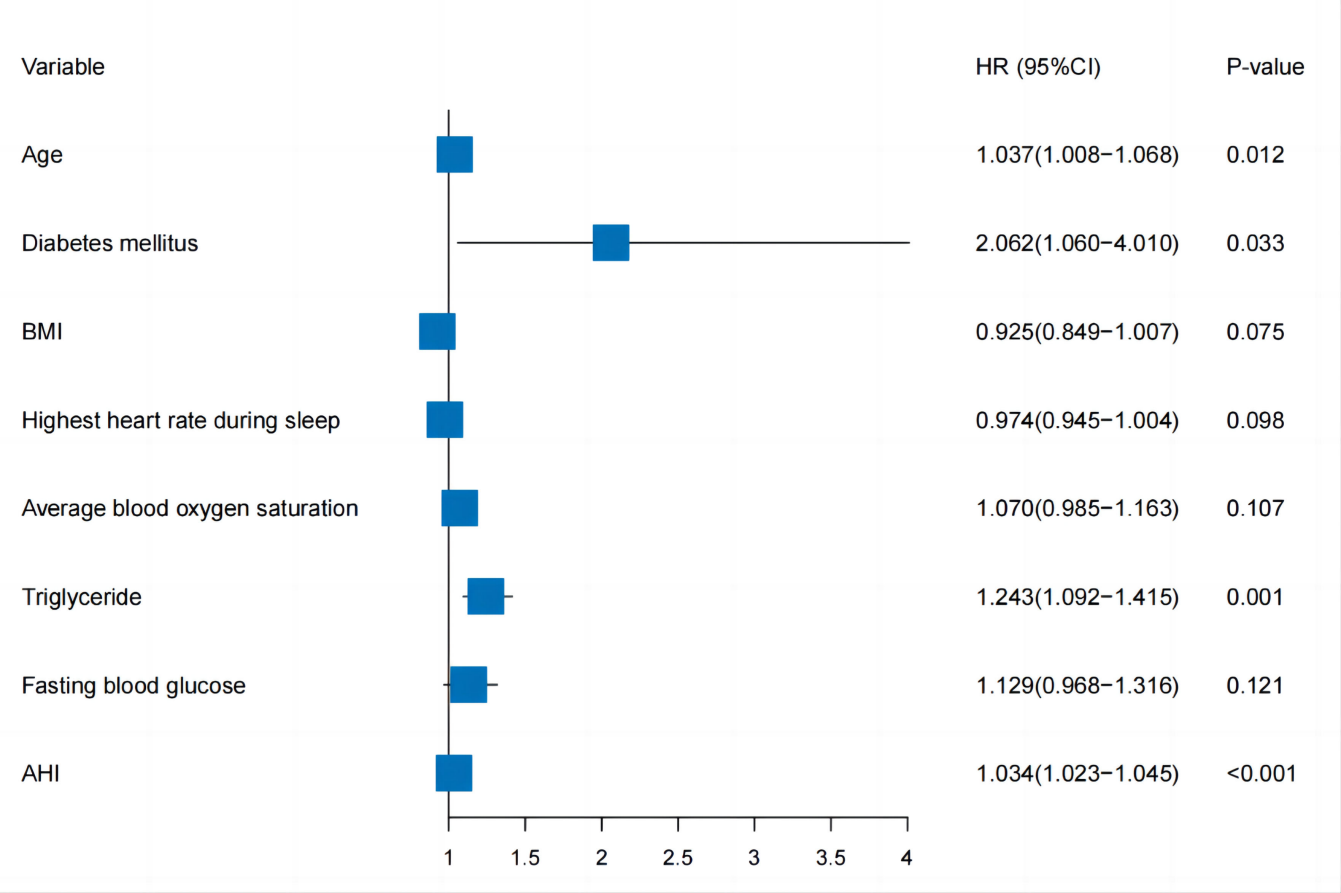
**Forest plot of the parameters in the multivariate Cox regression analysis.** BMI, bady mass index; AHI, apnea-hyponea Index; HR, hazard rate.

**Fig. 4.**
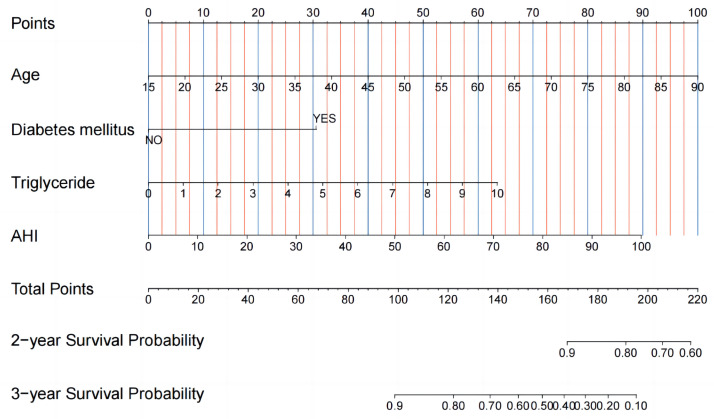
**A prognostic nomogram has been developed employing independent predictive factors.** To determine a patient’s score, the vertical line corresponding to each clinical feature in the top row is followed, and the points for four features are then added together to obtain the total score, which is displayed in the middle row. The 2- and 3-year survival probability can be determined by drawing a vertical line across the bottom two rows, based on the total score. AHI, apnea-hyponea Index.

LASSO regression analysis along with 10-fold cross-validation identified the following nine potential predictors of MACCEs, including diabetes mellitus, triglyceride, AHI, fasting blood glucose, age, apnea maximum duration, diastolic blood pressure, highest heart rate during sleep, and body mass index (Figs. 1,2). Each variable had a different correlation coefficient strength, as presented in Fig. 3. A multivariable Cox regression analysis was conducted to evaluate the adjusted effect estimates on MACCEs associated with systemic arterial hypertension patients with suspected OSA, which identified age (hazard rate, HR: 1.037, 95% CI: 1.008–1.068, *p* = 0.012), diabetes mellitus (HR: 2.062, 95% CI: 1.060–4.010, *p* = 0.033), triglycerides (HR: 1.243, 95% CI: 1.092–1.415, *p* = 0.001) and AHI (HR: 1.034, 95% CI: 1.023–1.045, *p* < 0.001) (Fig. 3). After implementing these four independent and valid variables, a prognostic nomogram for systemic arterial hypertension risk stratification at 2 and 3 years was constructed (Fig. 4).

### 3.3 Performance of the Prediction Nomogram

We assessed the efficacy of the prognostic nomogram in tracking the discriminatory power of MACCEs, implementing the Harrell’s C-index and computing the area under the receiver operating characteristic (ROC) curve (AUC). The Harrell’s C-index for separating MACCEs and MACCEs-free cases in the training cohort was 0.773, 95% CI (0.718–0.828).

**Fig. 5.**
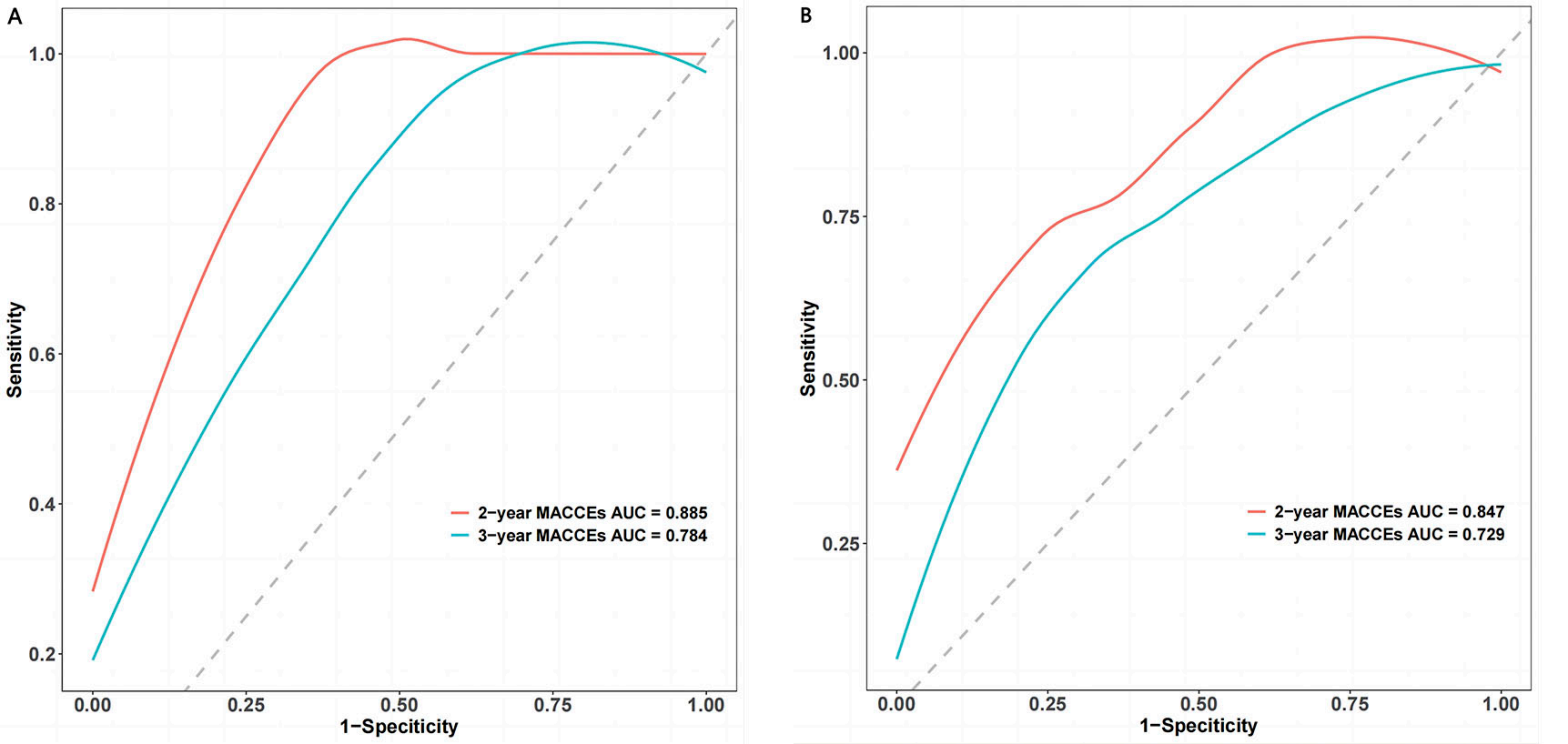
**Receiver operating characteristic (ROC) curve analysis was conducted to evaluate the discriminative ability of the prognostic nomogram in predicting the 2-year and 3-year MACCEs risk in both the training (A) and verification cohorts (B).** MACCEs, major adverse cardiac and cerebrovascular events; AUC, area under the ROC curve.

Additionally, the verified cohort had an equivalent Harrell’s C-index of 0.78 (95% CI: 0.678–0.882). This model has demonstrated remarkable predictive accuracy for 2-year and 3-year MACCEs, boasting an impressive area under the ROC curve of 0.885 and 0.784 in the training cohort, respectively. Furthermore, in the verification cohort, the nomogram model continued to exhibit strong discriminatory power, with an area under the ROC curve of 0.847 and 0.729 for 2-year and 3-year MACCEs, respectively (Fig. 5A,B).

**Fig. 6.**
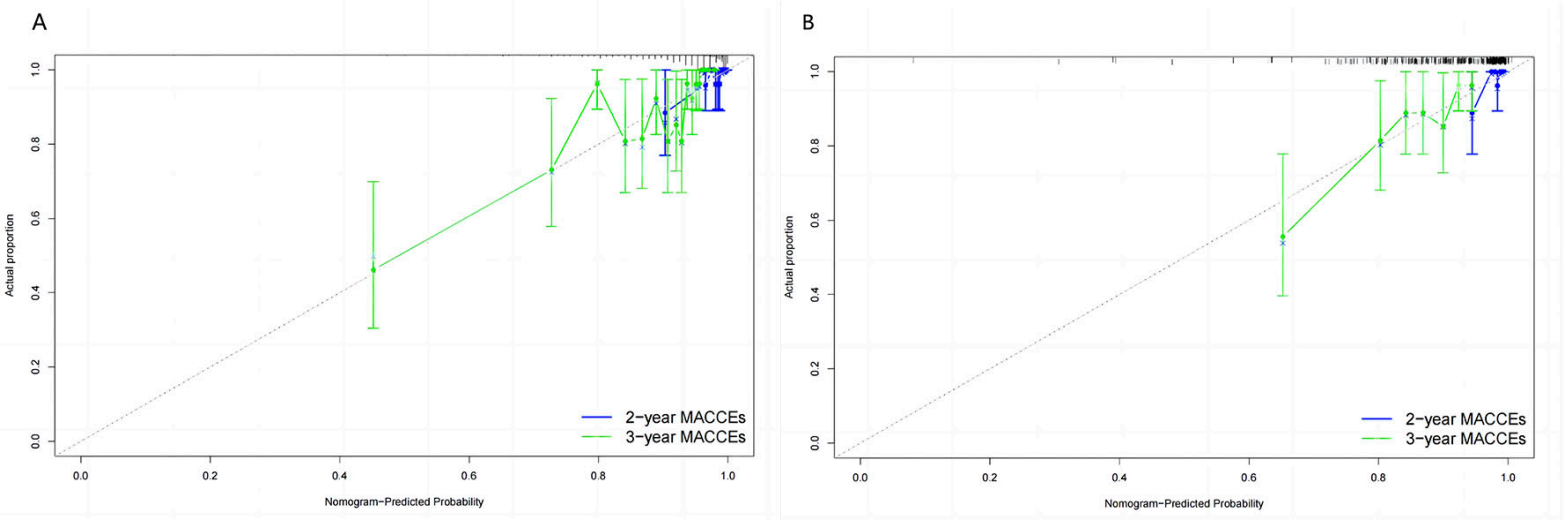
**Calibration of a prediction nomogram for the discrimination of MACCEs and MACCEs-free survival in the training (A) and the verification cohorts (B).** MACCEs, major adverse cardiac and cerebrovascular events.

After determining the MACCEs risk captured by the prognostic nomogram, we evaluated the degree of deviation between predicted and observed clinical outcomes through a calibration plot. The prognostic nomogram exhibited significant discriminatory ability and consistent performance in predicting 2- and 3-MACE risk across both the training and verification cohorts (Fig. 6A,B).

## 4. Discussion

We sought to validate the performance of a novel tool quantitative risk-stratified for systemic arterial hypertension patients with suspected OSA, generated from a prediction nomogram trained on multimodal data in cohort, to capture MACCEs risk in a Chinese population. The prediction models demonstrated excellent performance in the internal validation cohort. The prognostic tool was validated using integrated multimodal data, which included demographic features, laboratory test parameters, and sleep monitoring data. This allowed for accurate forecasting of MACCEs in hypertensive patients with suspected OSA. Furthermore, this thorough evaluation and predictive approach could enhance the accuracy of diagnostic and therapeutic strategies for systemic arterial hypertension and OSA, ultimately conducive to an improvement in a patient’s quality of life and overall health.

Nomogram models of multimodal data integration have the potential to accurately predict poor outcomes among cardiovascular disease patients. This integrated approach expands the pool of informative characteristics beyond traditional risk factors to include clinical text data, biomarkers, and imaging features, thereby enhancing the predictive ability of the model [5, 6, 19, 20]. The multimodal nomogram models, which we recently adapted from previous studies, have demonstrated excellent performance in accurately classifying individuals as either OSA cases or controls [6, 17]. Nonetheless, there is currently limited evidence in the literature to demonstrate that established probability scores derived from nomogram models can serve as a reliable quantitative marker for systemic arterial hypertension patients with suspected OSA who are at risk of developing MACCEs. Notably, given the current spread and changing trends in systemic arterial hypertension merging with OSA in China, its magnitude should be fully perceived by everyone [15, 16]. Previous literature reveals that AHI exists as a continuous phenotype and reflect that different degree of OSA, even subclinical changes, confer different degrees of risk for worsening prognosis [21, 22, 23]. Undeniably, the instance of OSA can result in a vicious cycle of worsening control and worsening systemic arterial hypertension driven by inflammation and metabolic disturbance [24]. If it is not detected early and treatment is not timely, it may trigger more grave consequences. As a consequence, the combination of systemic arterial hypertension and OSA is a “low-risk” state, a thorough evaluation of the patient’s individual condition is conducted, accurate predictions of disease progression are made, and dynamic warnings are issued in a timely manner to predict so as not to bring irreversible harm to individuals and society.

Concurrently, as stated in previous literature, age, diabetes mellitus as well as triglyceride levels were well-established and shared risk factors for systemic arterial hypertension and OSA [18, 24, 25, 26, 27]. This nomogram model summarizes a continuum that demonstrates a quantitative increase in the risk stratification of systemic arterial hypertension patients with suspected OSA, which is based on the combination of AHI, age, diabetes mellitus and triglyceride.

Moreover, this finding has a constructive bearing on clinical precision in the management of systemic arterial hypertension, which is conducive to improving upon the “one-size-fits-all” approach and applicable to most hospital settings, even primary hospitals in China. Furthermore, our data explores the further approach to consider the heterogeneity of systemic arterial hypertension patients with suspected OSA, reflected by varying risk factors, pathophysiological causes and consequences. Additionally, emerging studies the method applied here, have taken advantage of this heterogeneity using analytic approaches such as multimodal data integration analysis to identify cardiovascular disease phenotypes, or subtypes of patients with unique characteristics, that may enable more personalized approaches to prognostication and treatment [2, 3, 4, 5, 6, 7].

The influence of OSA on cardiac structure and function is proportionate to the severity of the AHI [28]. Our data suggest that some individuals with systemic arterial hypertension, who did not have a diagnosis of moderate to severe OSA, showed clinical evidence of MACCEs. Furthermore, in line with previous studies, our findings confirm that age [29], diabetes mellitus, and triglyceride levels are independent risk factors for MACCEs. Particularly, age, a non-modifiable risk factor, correlates with an increased risk of MACCEs, due to the progressive nature of cardiovascular and cerebrovascular diseases [29]. Diabetes is a well-established risk factor for MACCEs. Individuals with new-onset diabetes present a 27% increased risk of developing cardiovascular disease and a 30% increased risk of experiencing myocardial infarction, compared to those without diabetes [30, 31]. Hypertriglyceridemia, or elevated triglyceride levels, also amplifies the risk of MACCEs [32]. High triglyceride levels are associated with atherosclerosis, a condition characterized by arterial hardening and narrowing, thereby predisposing individuals to cardiovascular and cerebrovascular events [32]. It is crucial to acknowledge that these factors often interact in complex ways. A quantitative assessment of these diseases can provide a more precise depiction of their phenotype, thereby facilitating improved risk stratification and enabling personalized diagnostic and therapeutic strategies.

On the whole, this model has the potential to provide a more accurate and personalized prediction of potential risks, leading to a more preventative and participatory future for managing systemic arterial hypertension patients with OSA. By tailoring treatment to individual patients, this approach not only leads to better patient outcomes but also significantly reduces the cost of diagnosis and treatment.

Implementation of quantitative risk assessment for systemic arterial hypertension has been facilitated by tools and direct electronic health record integrations that make risk estimates accessible for counseling and shared decision-making for systemic arterial hypertension prevention. Especially, as previously discussed, the implementation of a prediction nomogram for precision medicine requires a standardized set of characteristics across various health systems, access to multimodal data integration for research and advancement, and the necessary infrastructure and resources for evaluation and deployment in clinical settings. To ensure widespread adoption by health systems and clinicians, transparent and explainable models, such as those exemplified by visualized and characteristic importance analyses, are essential. Additionally, using this approach, accurate predictions of disease progression can be made, and timely warnings can be issued to prevent irreversible harm to individuals. Furthermore, this is a simple prediction nomogram tool that is widely available at low cost, allowing non-invasive risk stratification in new-onset systemic arterial hypertension patients with suspected OSA, while optimizing the diagnostic and therapeutic strategies of disease and shared decision-making for systemic arterial hypertension prevention.

## 5. Study Limitations

First, it is important to note that retrospective designs are unable to eliminate selection bias. As a result, it is imperative to integrate additional clinical evidence from other trials to further fortify our model. Second, our sample size was limited, and therefore, additional follow-up and a larger sample size are necessary to validate the long-term efficacy and generalizability of our findings. Third, this prediction model has not yet undergone external validation. Therefore, future studies will need to demonstrate its applicability in other populations.

## 6. Conclusions

We leveraged a multimodal data integration model trained on longitudinal cohort to synthesize a quantitative risk stratification for hypertensive patients with suspected OSA. This model predicts which individuals may have a potential high risk for MACCEs at an early stage. Further research in prospective studies is required to assess the association of prediction models with the incidence MACCEs, and to examine its efficacy in other populations.

## Data Availability

Individual participant data that underlie the results reported in this article, after de-identification can be obtained from the corresponding author upon reasonable request.

## Supplementary Material

Supplementary material associated with this article can be found, in the online version, at https://doi.org/10.31083/j.rcm2507258.
