# Supplementary material for: Multimodal Data Integration Enhance Longitudinal Prediction of New-Onset Systemic Arterial Hypertension Patients with Suspected Obstructive Sleep Apnea
Source: Rev Cardiovasc Med. 2024 Jul 10;25(7):258. doi: 10.31083/j.rcm2507258 (PMC11317349; doi:10.31083/j.rcm2507258)
Supplement: Supplementary file 1 [file 2153-8174-25-7-258-s1.doc]

**Supplementary Material.** **Inclusion and exclusion criteria.**

| **Inclusion criteria** |
| --- |
| Low to intermediate pretest probability for obstructive sleep apnea |
| Successful polysomnography screening |
| New-onset systemic arterial hypertension |
| Patients exhibiting pronounced snoring, excessive daytime sleepiness, or observed instances of nocturnal apnea were categorized as being at a suspected obstructive sleep apnea |
| Follow-up was completed. |
| **Exclusion criteria** |
| Acute coronary syndrome |
| Chronic or acute cardiac failure |
| Acute- and chronic-phase inflammatory responses |
| Depressed left ventricular systolic function (ejection fraction <30%) |
| Malignant tumor |
| Dilated cardiomyopathy |
| Rheumatic heart disease |
| Myocarditis or cardiomyopathy |
| Infectious or severe liver or kidney disease |
| Patients without the results of a sleep monitoring |
| Lost to follow-up. |
